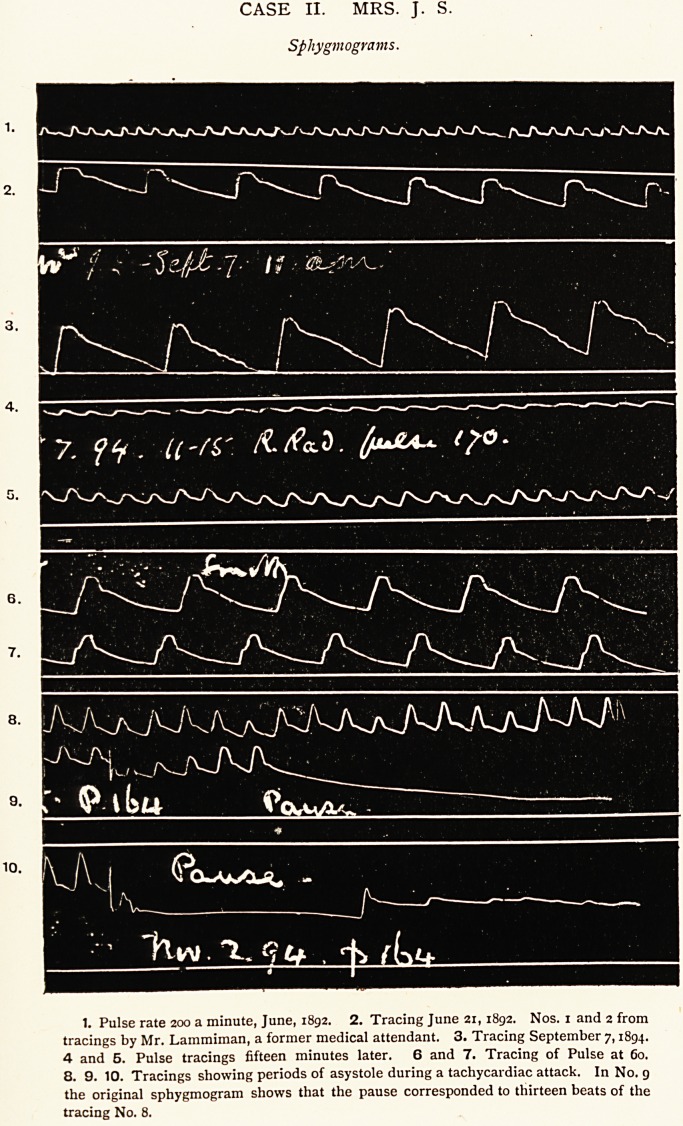# Paroxysmal Tachycardia

**Published:** 1897-06

**Authors:** P. Watson Williams


### PAROXYSMAL TACHYCARDIA.

BY

P. Watson Williams, M.D. Lond.

The cases comprised under the term paroxysmal tachycardia form a clinical entity characterised by attacks of greatly increased frequency of the heart-beat, varying from 130 to 300 a minute, of sudden onset, recurring at long or short intervals and of variable duration.

Paroxysmal tachycardia appears to have been first described by Payne Cotton[1] in 1867; Sir Thomas Watson[2] and Dr. Bowles[3] reported other cases immediately after Payne Cotton's recorded case. Since that time the condition has been frequently observed, and has been the subject of much discussion.[4] A consideration of these cases, and of those observed by myself, leads to this conclusion, that while gross pathological conditions are sometimes associated with the paroxysmal attacks of tachycardia, the latter affection is a functional disturbance, the precise pathology of which is as yet undetermined. The theories advanced in explanation of the phenomena are, briefly, that this disease is due: (1) To excitation of the accelerator nerve of the heart; (2) to paralysis of the vagus; (3) to disease of the intracardiac ganglia; (4) to disease of the myocardium. These various views will be examined further on; but it is evident that while such opposing theories are put forth in explanation of a disease, we are very much in the dark as to its true pathology. So many difficulties have to be faced in seeking to find a solution of this interesting pathological problem, that I have thought it best to consider that aspect of my subject after discussing the symptoms and etiology.

[1] *Brit. M. J.*, 1867, i. 629. [2] *Ibid.*, 752. [3] *Ibid.*, ii. 53.

[4] Since writing my paper, Dr. W. P. Herringham has published in the *Edinburgh Medical Journal* for April an interesting article on the affection, with an analysis of fifty-three recorded cases, and I have not hesitated to avail myself of his observations and bibliography in amplifying my own notes on the literature.

*Symptoms.*—The attacks supervene without any apparent cause in the majority of cases, though in others they follow on strong emotion or fatigue, or are associated with dyspepsia. The onset is always abrupt and sudden; the heart-beats do not increase in frequency progressively, but immediately pass from the usual rate to the extreme frequency of tachycardia, attaining a rate of from 150 to as much as 300 per minute.[1] Curiously enough, it is only rarely that the patients perceive any subjective sense of palpitation, though as a rule they are conscious of the attack from a peculiar sense of tremor and lassitude, with a feeling perhaps of discomfort in the precordial region, of carotid pulsation or constriction in the throat or chest.

The attacks generally cease as suddenly as they come on, the cessation being accompanied by a feeling of "a violent blow on the nose" (Case 2), "a rebound in the whole body" (Œttinger's case[2]), "a sense of impending death, or as if something had burst in the cardiac region" (Bouveret[3]), or a general sense of relief and well-being. The attacks are of variable duration, lasting from a few seconds to some weeks, recurring at intervals of months or years, in most cases, but with much greater frequency in some cases. During the short attacks, the physical signs are little marked; the pulse is small, frequent, regular, and of low tension; the carotids pulsate, and the apex impulse is more plainly seen and felt than usual. In the intervals between the attacks, the heart is usually normal in rhythm and function.

But when the attacks persist for many hours or days, signs of cardiac failure supervene, in the form of pallor, soon giving place to cyanosis, dyspnœa, pulmonary œdema, bronchorrhœa with copious mucous expectoration and dyspnœa, anasarca, oliguria; sleep becomes restless, or the patient may suffer from insomnia, and sometimes cerebral disturbances arise, such as nightmares, delirium, &c. It is unusual, and probably only accidental, for organic disease of the heart to be present in these cases; but in the long attacks the heart becomes consider-

1 Bristowe records a case in which he counted a pulse of 308 a minute, *Diseases of the Nervous System*, 1888, p. 123.

2 *Med. Week*, 1894, ii. 470.

3 *Rev. de Méd.*, 1889, ix. 753.

ably dilated and mitral incompetence arises, the liver is enlarged, the jugulars pulsate, and transient albuminuria may supervene.

The two cases I will now relate are fair types of the mild and aggravated forms.

Case 1.—Mr. A. P., aged 81, had suffered from paroxysmal tachycardia from boyhood, and could perfectly well recall attacks as far back as 1843 (fifty-three years). His mother had suffered from similar attacks. His pulse would suddenly rise to 120 or 130 a minute, and as suddenly drop again to 60. In his old age it occurred less frequently than when he was young.

He could not give any cause for the attacks, but stated definitely that they were associated with dyspepsia, and he believed indigestion induced the attacks. During an attack he felt no great discomfort, but it was often accompanied by a "feeling of compression over the root of the nose." The attacks sometimes lasted a few minutes only, sometimes many hours, and they gave rise to a sense of lassitude; but he never felt any active sense of palpitation. The attacks were more liable to come on while he was resting. He had no difficulty or oppression in breathing in an attack. He had found by long experience that a strong nip of brandy was the best thing to cut short an attack, and that if that was not sufficient, "digitalis and brandy together invariably controlled the condition."

Case 2.—Mrs. J. S., aged 62, consulted me in August, 1894, and was subsequently seen with me on many occasions by Dr. Long Fox and Dr. Shingleton Smith. She complained of intense weakness, and had a cough with rather profuse muco-purulent expectoration. She also stated that she was subject to attacks of palpitation.

The patient stated that when about three months old she had inflammation of the lungs, and that she believed she had never recovered from the effects of that attack. At any rate, she had always had a delicate chest and had been subject to chronic bronchitis from early childhood. Twenty years ago, she remembered the cough with expectoration was very troublesome and it had gradually increased, but had been greatly aggravated ever since her heart-attack last winter.

Her first heart-attack came on after a long and weary journey from Madeira, about six years before I saw her. The onset was sudden, and seemed "like an engine in her chest"; it lasted ten minutes only, stopping as suddenly as it came. She felt utterly prostrate from it and thought she would die. Six or seven months later she had her second attack, and then she had attacks about every six months, each lasting a few hours. In 1892 she had attacks, and one lasting as long as seventeen hours, the pulse reaching 200 a minute. Not long after, she told me, she had consulted Sir Douglas Powell for her chest, and that she had a heart-attack while in his study, when the pulse-rate was 240. From that time her attacks recurred at frequent intervals. In the spring of 1894 she had a very long attack, lasting a "whole week," while under Dr. Thomas, of Southampton. The attacks, as I observed them, will be described further on.

When I saw her she was very thin—in fact, emaciated, suffering from gastric catarrh. Loud râles could be heard all over the chest, front and back, and though there was no area of dulness on percussion, distinct and well-marked cavernous breathing and voice-sounds were found over the base of the left lung.

The heart was somewhat dilated, the apex-impulse being in the 5th interspace, one and a half inches outside the nipple line. The pulmonary second sound was accentuated, but no bruit was present at that time. The pulse-rate was generally between 44 and 48 a minute, but it often varied from one minute to the next, reaching 50 to 60 a minute, and was often as low as 40.

She was a bright, intelligent lady of neurotic temperament, but with no evidence of any tendency to hysteria. On August 30th she had one of her "attacks." She said she could generally tell beforehand when an attack was about to come on, but could not describe any definite prodromal symptoms beyond an unusual sense of languor. They came suddenly and left suddenly, often "with a sensation of three violent blows on the bridge of the nose." Sometimes an attack would be cut short by a strong nip of whisky. During an attack she generally felt tired and disinclined for any exertion, but as a rule felt no sense of palpitation. Respiration was not appreciably affected during an attack; but it must be remembered that when I saw her the lungs were extensively diseased, and the respiration-rate was generally 40 or 50. The pulse would often range as high as 200 a minute during the attack.

The following notes and the sphygmographic tracings well illustrate the variations in pulse-rate:—

| | | | | | | | | | |
|---|---|---|---|---|---|---|---|---|---|
| Aug. 30. | 10.30 a.m. | ... | pulse | 160 | Aug. 30. | 11.15 a.m. | ... | pulse | 112 |
| ,, | 10.45 ,, | ... | ,, | 156 | ,, | 4.15 p.m. | ... | ,, | 44 |
| ,, | 10.55 ,, | ... | ,, | 156 | ,, | 4.30 ,, | ... | ,, | 198 |
| ,, | 11.0 ,, | ... | ,, | 104 | ,, | 4.32 ,, | ... | ,, | 204 |

Six cactina pellets, with fifteen minutes' interval between each, did not seem to relieve her, but were followed by excitement and wandering. One-fifth of a grain of morphine given in the evening apparently gave rise to persistent vomiting, without any relief.

On Oct. 20th, at four p.m., Dr. Shingleton Smith saw her with me during an attack which had lasted since the previous day. The pulse-rate was 200 a minute. Although she had had very frequent attacks, and the one she was in at the time we saw her had lasted nearly twenty-four hours, there was no cardiac bruit, nor was the pulse affected by sitting up. The liver dulness was not increased. We gave her syr. chloral ʒi., tinct. dig. ♏x., sp. æth. co. ♏x., every two hours.

She had six doses of the mixture, yet passed a sleepless night; but on the following morning the pulse had improved in volume, though still small and of low tension and down to 48 a minute. On Oct. 22nd, the pulse having meanwhile improved and ranging about 64 a minute, a distinct mitral systolic bruit was audible. During November and December, in my absence from home, Dr. Shingleton Smith kindly took charge of the case. She had several attacks, but the heart in some measure seemed to be controlled by infusion of digitalis or by digitalin.

In January she had become worse, and on Jan. 8th she lost consciousness and was apparently dying; but this condition was probably unconnected with her heart-trouble, and the following day she was better again. On Jan. 10th, while I was feeling her pulse, which was 60 and fairly strong, it *suddenly* became very feeble and irregular: 120 beats a minute could be counted with difficulty. Auscultating the heart at once, I found it was beating regularly at 200 a minute. A few minutes later, whilst my finger was on the pulse, it *suddenly* reverted to 60 a minute, becoming at once regular and fairly strong again.

It would be tedious to follow the case in detail any further. Suffice it to say that the condition of the patient, till the time of her death on Sept. 8th, 1895, was practically a repetition of the varying conditions just described.

Atropine, cactus, chloral, chloralamide, and sparteine sulphate had all been tried in considerable doses, but none of them had any appreciable effect in controlling the heart condition. Digitalis frequently failed, but on the whole I believe it materially helped the cardiac condition, especially when signs of failing circulation supervened after prolonged attacks. Yet it did not prevent the occurrence of the tachycardia, nor can one say that it cut short any attack, for the attacks were so variable in duration that it was impossible to determine how far their cessation was attributable to the influence of the drug. The patient died suddenly in the night, when she had been apparently going on as well as usual.

Dr. Shingleton Smith and Dr. Fisher made a *post-mortem* examination, as I was away from home. The "cavity" at the base of the left

### CASE II. MRS. J. S.

*Sphygmograms.*

1. 2. 3. 4. 5. 6. 7. 8. 9. 10.

**1.** Pulse rate 200 a minute, June, 1892. **2.** Tracing June 21, 1892. Nos. 1 and 2 from tracings by Mr. Lammiman, a former medical attendant. **3.** Tracing September 7, 1894. **4** and **5.** Pulse tracings fifteen minutes later. **6** and **7.** Tracing of Pulse at 60. **8. 9. 10.** Tracings showing periods of asystole during a tachycardiac attack. In No. 9 the original sphygmogram shows that the pause corresponded to thirteen beats of the tracing No. 8.

lung was found to be a group of finger-like dilated bronchial tubes in the collapsed lower lobe; all other parts of the lungs were emphysematous. The heart was large and flabby, the muscle pale and soft; it was considerably dilated, and the mitral valve incompetent. The stomach was dilated, but otherwise appeared normal.

*Etiology.*—My first patient stated that his mother suffered from paroxysmal tachycardia, and Œttinger's patient stated that his maternal grandmother, who died aged 96, and his mother, who died young in childbirth, had been subject to paroxysms of palpitation; while his son, aged 29, had for some years suffered from the same condition. It is worthy of note that the grandfather of Buckland's case[1] was a general paralytic, and that the grandmother died insane. The mother and brother of Faisans' patient[2] probably suffered from paroxysmal tachycardia.

In several cases the attacks have supervened after influenza. Dr. Symes Thompson,[3] at a discussion on influenza at the Medical Society of London, called attention to the records of neurotic disturbances during previous epidemics, and alluded to the theory that pulmonary affections were due to the removal of nerve-control of the lung, observing that there was a good deal of evidence of serious affection of the function of the vagus. In several cases he had met with tachycardia, the heart-beat reaching 200 a minute.

There was a history of rheumatic fever or rheumatic pains prior to the attacks in 7 out of 57 cases. Syphilis had been contracted in still fewer cases. One or two cases have followed after malaria, and one arose after measles. Oliver's case[4] had become very nervous after an accident, and it was after this extremely neurotic condition had developed that the attacks began to come on. One of Klemperer's patients[5] stated that the paroxysms had immediately followed upon a severe blow on the chest, and another developed the condition while in hospital with a compound fracture of the leg. In Talamon's case[6] the affection developed after a fall on the head. Seymour Taylor

1 *Tr. Clin. Soc. Lond.*, 1892, xxv. 92.

2 *Bull. et Mém. Soc. méd. d. Hôp. de Par.*, 1890, 3e sér. vii. 964; cited by Herringham.

3 *Brit. M.J.*, 1891, i. 1312. 4 *Ibid.*, 217.

5 *Deutsche med. Wchnschr.*, 1891, xvii. 335.

6 *Semaine méd.*, 1891, xi. 13; cited by Herringham.

suggests[1] that it is liable to arise reflexly from disease of the abdominal viscera. But in the great majority of cases no cause for the occurrence of the disease is assigned.

*Diagnosis.*—The abrupt onset and decline of the peculiar paroxysmal attack without any adequate apparent cause, and occurring in a heart generally healthy, is so characteristic that there is seldom any difficulty in differentiating the affection; for, as Œttinger aptly puts it, "all cases of essential tachycardia are, so to speak, cut from the same pattern."

There is no exophthalmos, enlargement of the thyroid, general tremor, or other symptoms, which go to make the syndrome of Graves's disease, apart from the fact that the onset and decline of the tachycardia of Graves's disease is less abrupt than in the affection under discussion.

Cases of injury to or disease of the vagus nerve, or degeneration of the bulbar nuclei of the inhibitory nerve of the heart, are often accompanied by abnormal frequency of the pulse, amounting to tachycardia; but the tachycardia in such conditions is persistent, not paroxysmal. The only cases which might be mistaken at first are those in which the patient is seen for the first time during the persistence of a long attack, with the physical signs of cardiac failure, with oliguria and albuminuria, especially if, as very rarely happens, there is evidence of concomitant organic heart disease. Even in these cases the history of previous attacks would be such as to lead to a strong suspicion of the true nature of the disease.

*Prognosis.*—Many writers appear to regard the disease as a purely functional disturbance of the heart, and it is often regarded more lightly than is warranted by a consideration of the cases recorded.

It may arise at any age; thus, at the Clinical Society, W. P. Herringham[2] recently exhibited a girl, who probably had attacks from the age of six years. The child was under observation from Sept., 1895, when she was eleven years old, to June, 1896, during which time she had seven attacks, and the history showed that she had been subject to similar attacks for at least five years. They began quite suddenly in a period of

[1] *Practitioner*, 1891, xlvii. 18. [2] *Brit. M.J.*, 1897, i. 144.

perfect health, and without adequate cause, the heart-beats reaching 260 a minute. The heart was permanently enlarged, and Herringham suggested there might be adherent pericardium, and that the myocardium itself might, as the result of some former disease, be unhealthy; but there was no further evidence of such disease.

Buckland has related[1] a very similar case in a girl aged 11, whose history pointed to the attacks having occurred at intervals from the age of 6. She had developed measles when Buckland saw her, and her temperature rose during the attacks sometimes to 103° F.

In the cases collected by Herringham 30 were men and 23 women. Seven patients were aged between 20 and 30 when first seen, and 5 others gave this decade as the date when the attacks began (6 men and 6 women). Eight patients were seen between 30 and 40 years of age, and in five others the disease had begun at this period (all men). Ten cases were either seen or reported to have begun between 40 and 50 years (5 males and 5 females). Over 50 years, Herringham[2] found only 3 cases (1 male, 2 females). This author found records of 7 deaths; Brieger's case[3] died at 33 with fibrosis of the wall of the left ventricle, Bristowe's[4] at 19 with dilatation only, Eccles's[5] apparently in late middle life. Three deaths occurred between 20 and 30. Between 30 and 40, four of the patients died, and there were two *post mortems* recorded. In one the heart was found much enlarged, there were hemorrhages in the epicardium, fatty degeneration of the myocardium, and vegetations in the auricular surface of the mitral valve.[6] In the second,[7] the case of a man aged 35, who had had fits of palpitation and breathlessness for three months, there was a patch of recent fibrosis, deep red in colour, in the wall of the left ventricle. Four deaths occurred between 40 and 50 years, and the one examined *post mortem* revealed only dilatation of the heart.

To these may be added the two cases I have recorded. The

1 *Loc. cit.* 2 *Loc. cit.* 3 *Charité-Ann.*, 1888, xiii. 193.

4 *Op. cit.*, p. 23. 5 *Lancet*, 1891, ii. 118. 6 Fränkel, *Charité-Ann.*, 1878, v. 273.

7 Fräntzel, *Deutsche med. Wchnschr.*, 1891, xvii. 321.

first, a male, had attacks in early boyhood, and died aged 81. The second, a female, apparently had her first attack at 56 years of age, and died aged 63. The *post-mortem* findings of the second case have already been described.

It will be seen that the prognosis must always be guarded; for although patients may be subject to attacks from early childhood and reach a ripe old age, the few *post mortems* recorded (eight) show that although there is very frequently nothing abnormal in the condition of the heart beyond obvious dilatation, the tendency of repeated and prolonged attacks is towards cardiac failure. Œttinger states that Bouveret, Sollier, and A. Fränkel have reported cases of death in the course of a paroxysm of tachycardia, the fatal issue being the result either of acute asystole or syncope, and there is little doubt that frequently recurring attacks may *per se* lead to a fatal result at a comparatively early age. Bouveret[1] observed a case in which the patient recovered after an attack lasting thirteen days; but Bristowe reports a case in which the paroxysm lasted five weeks, yet the patient recovered. No less than ten cases were observed by Bristowe, and he remarks that "speaking generally of these cases of recurrent palpitation, I should be inclined to say that the prognosis is fairly hopeful for those persons who are able to lead quiet lives, who avoid mental or bodily excitement and overwork,"[2] etc., and that the disease may even be arrested.

*Pathology.*—Paroxysmal tachycardia is so peculiar in the remarkable similarity of the cases as regards the essential features of the disease, that it is difficult to suppose that they are not all due to the same pathological condition, though the lesion may be induced by various causes. The mere fact that in a small proportion of cases there has existed evidence of organic disease of the heart can scarcely be held to support the view that it is due to disease of the myocardium. In most cases examined after death the heart has been found dilated; but while the dilatation and failure of the heart may have caused the death, it would be as illogical to attribute the paroxysmal attacks to dilatation as it is to say that disease of the

[1] *Loc. cit.* [2] *Op. cit.*, p. 135.

coronary arteries is the cause of angina pectoris, overlooking the fact that dilatation of heart and disease of the coronary arteries are both very commonly observed *post mortem*, while paroxysmal tachycardia and even angina pectoris are certainly uncommon in comparison.

Moreover, it is inconceivable that organic disease of the heart should give rise to these paroxysmal attacks from early childhood to old age without declaring itself, and that the attacks should recur at intervals, sometimes of months, sometimes of years, the heart affording no evidence of any abnormality in function in the intervals. I think we may therefore set aside the view that the affection is generally due to disease of the myocardium or pericardium, and assume that the hereditary history of several cases, the neurotic temperament of the majority of patients, the clinical character of the affection, and the usual absence of any evidence of organic heart disease point strongly in favour of the very general belief that paroxysmal tachycardia is a functional neurosis.

Cotton's original view was that the tachycardia was due to irritation by abnormal matters in the blood or to extraordinary sensitiveness of the heart. But the reports of other cases led him to see from the essentially paroxysmal nature of the attacks and their sudden onset and decline that toxic matters in the circulation would not account for the disease.

The theory that the affection is the result of paralysis or paresis of the vagus has been advanced by Tuchzek,[1] Nothnagel, and others. Nothnagel stated[2] that "it was conceivable that there might be . . . sudden paresis of the vagus," and that he was inclined to this view. "One might suggest that, in the same way that intermittent paralysis occurred in other motor nerves, such intermittent functional paralysis might also occur in the vagus." Now, Nothnagel believed that the fact that his patient could stop his attacks by a deep inspiration, which would put the terminations of the vagi in the lungs on the stretch, supported his pathological theory. But even if his explanation be correct, and we might cite with greater force the

[1] *Deutsches Arch. f. klin. Med.*, 1878, xxi. 102; cited by Herringham.

[2] *Brit. M. J.*, 1887, i. 238.

cases where compression of the vagi in the neck (3 cases) caused cessation of the attacks, it only showed that by strong stimulation the vagi were able to inhibit the motor ganglia in the heart, and it in no way proved that paresis of the vagus had caused the tachycardia. Simple pressure in the right ovarian region, as well as compression of one vagus, or electrisation of the precordial region, would cause cyanosis and slow the pulse in Brieger's case; while on the other hand, in most cases no amount of pressure or electrical stimulation of the vagus was of the slightest benefit. The vagus paresis theory is supported by Farquharson,[1] and by Handfield Jones who from a review of cases remarks that "the associated symptoms point very decidedly to the implication of the vagi in the disorder; in 5, the breathing was notably affected, and in 2 the stomach was deranged. My notion is that the pathema is a paralytic neurosis of the vagi, or of their cardiac branches, essentially similar to a common neuralgia, *e.g.* sciatica."[2] The vagus has been examined histologically in one case at least (Brieger's case), and found absolutely normal. We have therefore to presuppose a structurally normal nerve is subject to intermittent paresis; for in many cases the pulse-rate is insufficient for a complete paralysis of the vagi, and yet no amount of stimulation by mechanical means, faradisation or drugs, suffices to increase its activity, which is nevertheless spontaneously resumed on the abrupt cessation of the attack.

Nor are we left to mere conjecture as to the result of paralysis of the vagus. In several cases of bulbo-spinal disease (bulbar paralysis, tabes,) the vago-accessory nuclei have been found atrophied, with consequent paralysis of the vocal cords.[3] Now the nuclei of origin of the inhibitory nerve of the heart are very close to the nuclei of origin of the motor laryngeal nerves, and have been equally involved in the atrophic degeneration, yet only *persistent* frequency of the pulse

[1] *Brit. M. J.*, 1875, i. 770.

[2] *Studies in Functional Nervous Disorders*, 1870, p. 608.

[3] I cannot of course enter here into the debated question whether the motor nerves of the larynx have their nuclei of origin in the bulbar accessory or in the nucleus ambiguus, and my wording must not be taken to imply adherence to either view.

has been observed, and the tachycardia has never been either paroxysmal or so rapid as has been observed in paroxysmal tachycardia. Gowers cites[1] a case of Guttmann's, in which a boy after diphtheria suffered from paralysis of the soft palate and one sterno-mastoid, and in whom the respiration became 12 and the pulse 120, and remarks that in many other cases a similar change in the pulse and respiration has been noted, and even a pulse-rate of 160 to 200. It is worthy of note, however, that Ross writes "there is no trustworthy record of retardation of the pulse which could with probability be referred to irritation of the vagus, but a pulse rising before death from 130 to 150, or even higher per minute, has been frequently recorded, and is probably caused by paralysis of the vagus."[2] Many cases of division of one vagus nerve by injury or during surgical operations are collected by Roswell Park,[3] and he states that from "studying case-histories, however, we come to the conclusion that the equality and frequency of the pulse is practically uninfluenced by pneumogastric division, and that no really serious disturbance has ever been really due to such accident." In cases of profound poisoning by atropine the pulse in man may be doubled, atropine paralysing the terminations of the vagi in the heart. A very interesting case is recorded by Gaipa and Titone,[4] in which the respirations in a strong man, aged 25, for a time became quickened after influenza, amounting to 120 a minute. They attributed this to a temporary functional disorder of the respiratory centre in the medulla (and perhaps Symes Thompson's cases of tachycardia after influenza were somewhat analogous,) but there was no history of the attack being paroxysmal. At any rate, this case would seem to show that it is possible for an isolated lesion of the cardiac inhibitory nuclei to occur; consequently one of the many difficulties in accepting the vagus theory would be removed. Yet, although some cases of tachycardia may be rightly attributed to a central medullary lesion, I can

[1] *Diseases of the Nervous System*, 2nd ed., vol. ii., 1893, p. 280.

[2] *Diseases of the Nervous System*, 2nd ed. 1883, vol. i. p. 975.

[3] *Ann. Surg.*, 1895, xxii. 145.

[4] *Riforma med.*, 1891, vii. pt. 4. 688; abstract in *Brit. M. J.*, 1892, i. Epitome, p. 33.

discover no sound reasons for referring *paroxysmal* tachycardia to a lesion of the medulla or of the vagus nerves.

There are still stronger grounds for rejecting the view that paroxysmal tachycardia arises from excitation of the augmentor nerve of the heart. I may remind the reader that the augmentor nerve-fibres are derived from the spinal cord by small medullated fibres, which pass out from the anterior nerve-roots of the upper dorsal nerves, especially the second and third, to reach the cervical sympathetic ganglia. These fibres passing through the sympathetic ganglia form the cardiac branches of the sympathetic to the superficial and deep cardiac plexuses. Now, with the inhibitory nerves intact, it is apparently impossible to obtain a greater rate of cardiac beat than 150 by any stimulation of the augmentor nerves, and moreover "in the mammal when the augmentor fibres are stimulated, some time elapses before the maximum effect is witnessed, and the influence of the sympathetic may last some considerable time after stimulation has ceased" (Landois and Stirling[1]); and, further, not only is the rate of the heart-beat increased, but likewise the force. Thus even if in paroxysmal tachycardia we conceive that the vagus is paralysed or paretic, while the sympathetic cardiac nerves are stimulated, we are still confronted with the clinical fact so constantly observed in paroxysmal tachycardia that the cardiac beats pass abruptly from the normal, or even abnormally slow rate, to the very rapid rate of 180, 200, or even 300, and as abruptly return to the normal. And yet if the theory I hold as to the pathology of Graves's disease be rightly held, we have already clinical examples of paresis of the vagus with excitation of the sympathetic. The medulla oblongata contains "many centres presiding over complex co-ordinated organic phenomena, regulating and controlling in their mutual action and reaction still lower organic centres in the spinal cord, in the heart muscle, in the muscular walls of the vessels, etc. But it is inconceivable that, between the lower organic centres in the medulla and the highest perceptive and volitional centres in the cortex, there are no intermediate or organic centres

[1] *A Text-Book of Human Physiology*, 4th ed. 1891.

higher than those in the medulla, viz. subconscious-emotional or higher organic centres, which regulate and control the very complex organic phenomena of emotion. . . . If such higher organic centres exist they are subject to disease, and while on the one hand their disturbance will be attended by the highly complex organic phenomena corresponding to those normally associated with profound emotional disturbance, they will be accompanied by the still less understood conscious phenomena of emotion conveniently described as emotivity. Now sudden intense or prolonged shock or grief in the first instance inhibits the heart's action, slows the pulse, raises arterial tension, inhibits secretion of the skin, etc. But if sufficiently intense, or if acting on an inherently weak nerve structure, such a degree of molecular disturbance may be induced in the affected regions that death may be caused either immediately or in a short period; or if the disturbance is less marked, the affected nerve structures may be rendered more or less permanently paretic, with consequent secondary phenomena of emotional shock, viz. tachycardia, arterial dilatation with low tension, sweating, etc. Thus, from defective control, the accelerator nerve to the heart runs riot on the slightest provocation, while flushing, perspiration, and excitation are exaggerated by slight causes, such as the visit of a stranger, slamming of a door, and so forth, while, together with exophthalmos, ocular paresis, and thyroid enlargement, they are more or less constantly present. This group of symptoms is known as Graves' disease. The excessive thyroid secretion, when present, aggravates the symptoms which it did not cause, just as starch and sugar in the diabetic diet aggravates the disease which these common articles of food do not cause."[1]

Another theory is that paroxysmal tachycardia is due to reflex excitation of the augmentor nerve of the heart. It is needless to recite again the discrepancies between the clinical facts of the disease in question and the known physiological phenomena which supervene on reflex excitation of the augmentor nerves, but I would draw attention to recorded cases in which reflex causes of irritation have given rise to tachycardia,

[1] *Clin. J.*, 1895, vii. 96.

an excellent example being furnished by Spencer Watson's[1] case of nasal polypi associated with tachycardia, which disappeared on removal of the polypi by snare and cautery. Dr. Sansom, he remarks, has met with similar cases, and on seeing this patient at once recognised the probable connection of the cardiac and nasal trouble. But in these cases the tachycardia is persistent and the pulse rarely rises above 150; in other words, the reflex tachycardia is sharply differentiated from paroxysmal tachycardia.

It only remains to consider the evidence in support of the view that in paroxysmal tachycardia we have to do with some pathological condition of the intracardiac automatic nerve centres. Physiological observations tend to prove that it is possible to obtain a much more frequent heart-beat by direct stimulation of the heart than by any known means of acting on the heart through the branches of the cardiac plexuses. Thus Frau Serafin,[2] by opening or closing, or by reversing, a strong constant current applied to the heart 140 or 180 times a minute, found the number of beats could be raised from 80 a minute to 140 or 180. It may be urged that this only proves that, uncontrolled by the inhibitory nerve, the cardiac muscle is capable of responding to this number of shocks per minute; but at any rate it shows that the intracardiac ganglia, unlike the extracardiac nerves, do not prevent this very frequent heart-beat. But it is certain that conditions may arise which render the intracardiac motor-mechanism independent of the inhibitory nerve; for instance, "physostigmin or Calabar bean excites the energy of the cardiac muscle to such an extent that stimulation of the vagus no longer causes the heart to stand still."[3]

Another noteworthy point in favour of the location of the neurosis in the intracardiac ganglia is the fact that *Cactus grandiflorus* always seemed to aggravate the attacks of my case, No. 2. Now Myers proved experimentally that in dogs, cactus or its active principle cactina directly stimulated the intracardiac accelerator ganglion, both vagi and sympathetic cardiac nerves

[1] *Brit. M. J.*, 1895, ii. 1097.

[2] Cited from Landois and Stirling, *op. cit.*, vol. i., p. 98.

[3] *Ibid.*, p. 99.

being divided, and to such a degree that stimulation of the divided end of the vagus no longer arrested the heart's action.[1]

Bearing these physiological data in mind, it is interesting and instructive to consider the clinical antithesis of tachycardia, bradycardia, and we shall find much to support the contention that the intracardiac ganglia are subject to functional disorders, which manifest themselves by definite clinical conditions. Hodgson[2] records a case of bradycardia with asystole in a man aged 44. The pulse fell steadily in the first three weeks after admission to the Sussex County Hospital from 108 to 52, and subsequently, after eleven weeks, to 24 per minute, when he developed pericarditis, from which he recovered. He was re-admitted twenty-seven weeks from his first admission, suffering from "fits" associated with syncope. When the pulse stopped for fifteen seconds, he was faint only; when it ceased for thirty seconds, an epileptiform fit occurred. For six months he was subject to these attacks, varying in number from one a week to a number daily so great as to be practically uncountable. On one day they occurred every quarter of a minute for ten hours. The average pulse at this time was 21, the slowest recorded pulse being 6. Latterly, the attacks were heralded by vomiting and a feeling of intense illness.

True bradycardia is not infrequently associated with vertigo and sudden loss of consciousness, which differs from an epileptic fit, and is the consequence and not the cause of the bradycardia;[3] in fact, the so-called fit is probably of the same nature as the sudden loss of consciousness in laryngeal vertigo.[4] Bradycardia is liable to abrupt onset and decline, as in Hodgson's case. It would almost seem that we may have bradycardia associated with paroxysmal tachycardia, for in my Case 2, as I have already mentioned, the pulse-rate, despite a very extensive lung trouble, was sometimes only 44; and the pulse tracings, Nos. 9 and 10, show very well the occurrence of prolonged asystole during attacks of tachycardia.

1 See my paper on *Cactus grandiflorus*, in *Practitioner*, 1891, xlvii. 266.

2 *Brit. M. J.*, 1891, i. 760.

3 On this point see Balfour, *Edinb. M. J.*, 1890, xxxv. 593.

4 See McBride's *Diseases of the Throat, Nose, and Ear*, 1892, p. 188.

For the reasons I have related above, I think that we are warranted in the conclusion that paroxysmal tachycardia is due to a neurosis of the intracardiac ganglia, and that although the affection is in many cases the outcome of concurrent pathological conditions affecting the coronary arteries, myocardium, or pericardium, it is most frequently a pure neurosis, which, as the result of prolonged or repeated attacks, is itself the cause of dilatation and functional disease of the heart.

Finally, I find that in arriving at this conclusion I practically accord with the pathological views of Herringham, who states that this localisation in the heart itself of any lesion that may exist is rendered probable from another point of view; viz., that of cases in which any original exciting cause is at all definite, such as running, going upstairs, stooping, lifting a heavy box, the cause is one which is likely to entail intracardiac pressure which might affect the intracardiac ganglia.

*Treatment.*—The results of treatment, on the whole, are eminently unsatisfactory in controlling or arresting the actual attacks. I have already remarked that in my severe case the only drug that in any way seemed to benefit the patient was digitalis, and that in the other case digitalis had given good results. Bouveret states that digitalis has proved of only moderate value. Œttinger keeping his patient in bed, found the pulse improved and the quantity of urine increased under digitalis; but Pye-Smith [1] has found digitalis and strophanthus "most disappointing in these cases. Absolute confinement to bed in the recumbent posture for a length of time led to slowing of the pulse, and often to complete cure." Yet my own experience and a consideration of recorded views of others tend to the conviction that although digitalis has but a limited action in controlling or aborting the actual attacks, it is the most useful drug for improving the circulation in the intervals between the severe and prolonged attacks. When the mitral valve has become incompetent from secondary dilatation of the heart, when the urine is deficient in quantity and albuminuria has occurred, when anasarca supervenes and the respiratory functions are embarrassed, then we shall find that digitalis in

[1] *Brit. M. J.*, 1891, ii. 1312.

some form affords the best chance of restoring the failing circulation, and improving the action of the heart generally.

Some have given morphine with advantage; at least, it sometimes calms the patient without cutting short the attacks. Oliver considered he had cured his patient with belladonna.

Caffeine, nitrite of amyl, and nitro-glycerine have been tried with no result. Sometimes a strong dose of brandy or whisky stops an attack, and in Nothnagel's case the attacks were arrested by deep inspiration.

In several cases faradisation of the vagi has been tried, and failed to have any effect; but pressure on the vagi in the neck, and in one case compression of the thorax, would stop the attacks. These procedures have been tried in other cases, however, and failed.

It is important to attend to the general health, and especially to rectify any gastric disorder; anæmia should be treated with iron and general tonics. Tea, coffee, smoking, undue exertion or excitement, and anything which tends to excite the nervous system, should be carefully avoided.

## Figures and Tables

**Figure f1:**
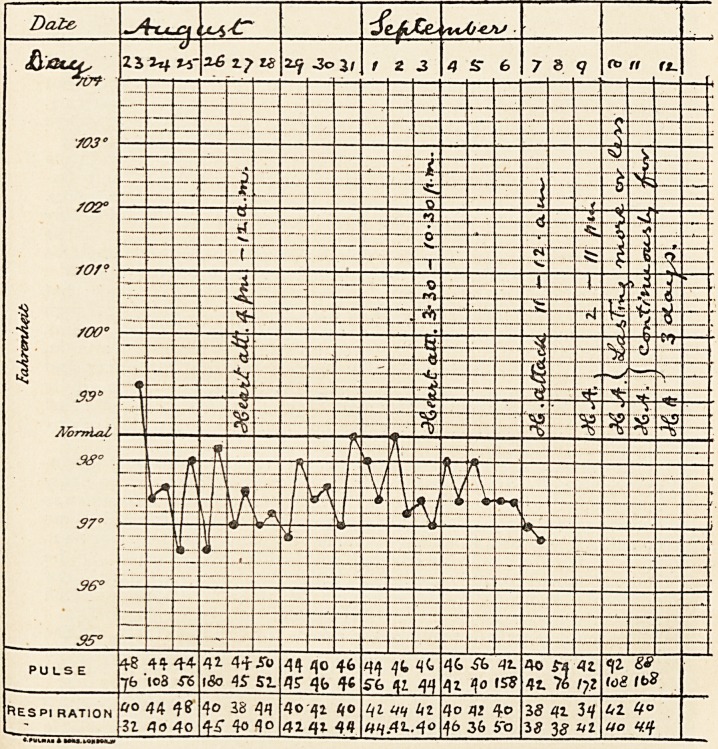


**CASE II. f2:**